# Surface Enhanced Raman Scattering for Biomolecular Sensing in Human Healthcare Monitoring

**DOI:** 10.1021/acsnano.4c15877

**Published:** 2025-02-27

**Authors:** Stacey Laing, Sian Sloan-Dennison, Karen Faulds, Duncan Graham

**Affiliations:** Department of Pure and Applied Chemistry, Technology and Innovation Centre, University of Strathclyde, 99 George Street, Glasgow G1 1RD, U.K.

**Keywords:** SERS, point of use testing, VOC detection, machine learning, wearable sensors

## Abstract

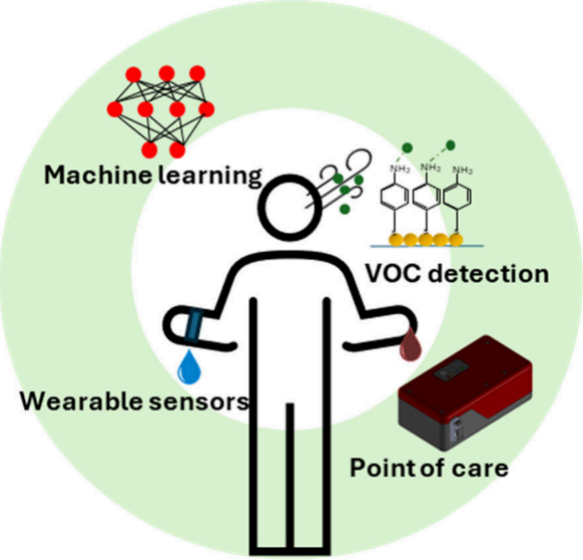

Since the 1980s, surface enhanced Raman scattering (SERS) has been used for the rapid and sensitive detection of biomolecules. Whether a label-free or labeled assay is adopted, SERS has demonstrated low limits of detection in a variety of biological matrices. However, SERS analysis has been confined to the laboratory due to several reasons such as reproducibility and scalability, both of which have been discussed at length in the literature. Another possible issue with the lack of widespread adoption of SERS is that its application in point of use (POU) testing is only now being fully explored due to the advent of portable Raman spectrometers. Researchers are now investigating how SERS can be used as the output on several POU platforms such as lateral flow assays, wearable sensors, and in volatile organic compound (VOC) detection for human healthcare monitoring, with favorable results that rival the gold standard approaches. Another obstacle that SERS faces is the interpretation of the wealth of information obtained from the platform. To combat this, machine learning is being explored and has been shown to provide quick and accurate analysis of the generated data, leading to sensitive detection and discrimination of many clinically relevant biomolecules. This review will discuss the advancements of SERS combined with POU testing and the strength that machine learning can bring to the analysis to produce a powerful combined platform for human healthcare monitoring.

## SERS for Biomolecular Sensing

In the 50 years since its discovery, surface enhanced Raman scattering (SERS) has been shown to be a powerful analytical technique in a variety of fields, such as biosensing, forensic science, environmental monitoring and food analysis.^1−4^ The wide applicability of the technique is owed to its sensitivity and ability to obtain specific molecular information from samples of low concentration, which is particularly useful in the detection of biomolecules. The capability of SERS for biomolecular detection was realized as early as 1980 when Cotton et al. demonstrated the use of the technique for the detection of cytochrome c and myoglobin.^5^ Although preliminary, the authors noted that the data was encouraging, and SERS should be used to solve bioanalytical problems. Throughout the 1980s, the direct, label-free detection of various biomolecules was achieved,^6,7^ again indicating the potential of the technique for sensitive detection in healthcare applications.

In 1989, the first labeled SERS immunoassay was developed, which illustrated the ability of SERS to be used as the analytical read out for the sensitive detection of biomolecules. Using SERS allowed the number of steps to be reduced by removing the need for washing in biological assays, shortening the time to results with no loss in sensitivity.^8^ In this work, the antibodies were labeled with a dye and adsorbed onto a silver (Ag) electrode for SERS detection. A later advancement introduced the functionalization of colloidal nanoparticles (NPs) with antibodies and a Raman reporter for incorporation into sandwich immunoassays and demonstrated that by using different Raman labels, multiple analytes could be detected simultaneously.^9^ To this day, SERS immunoassays are well studied and show excellent promise as a diagnostic test, where the incorporation of SERS nanotags can offer improved sensitivity and multiplexing capabilities over alternative detection methods.^10^

In parallel to the early protein-based detection, the development of the first SERS gene probe in 1994 highlighted the potential of SERS to detect DNA targets in biomedical applications.^11^ SERS was also employed for the discrimination of DNA from a low-concentration mixture without the need for separation, and for the simultaneous detection of multiple labeled oligonucleotides.^12,13^ DNA-based NP assays also emerged, where NPs were functionalized with a Raman reporter and specific DNA probes, such that SERS signals could be obtained upon binding with the target DNA.^14^

Each of these approaches for SERS-based detection of biomolecules has continued to be explored and developed and the advantages of each method have been realized for different biosensing applications. For example, label-free SERS detection can be applied to the analysis of biomolecules, pathogens, cells, tissues and biofluids, and has thus found use in many biological and biomedical applications.^15^ Due to the sensitivity of SERS and the molecule-specific spectra that are obtained, label-free detection provides an abundance of information at the molecular level, which can be linked to biological processes and health conditions, enabling detection and diagnosis of disease and treatment monitoring. However, there are some drawbacks, such as interference from other components of the sample, weak Raman signals from certain biomolecules, and that not all target molecules will have an affinity for the SERS substrate to allow enhancement of the inherent Raman signal. These issues can be overcome by using strongly enhancing SERS substrates, by introducing targeted biomolecule detection to ensure binding of the analyte at the surface of the substrate, or, alternatively, by incorporating a reporter molecule with a strong Raman signal, to further improve sensitivity. The latter can be achieved either by using a “Raman indicator” where the signal changes on interaction with the biomolecule, or by incorporating Raman tags with a distinct spectral profile. This approach also improves multiplexing capabilities as different labels with significantly different Raman spectra can be incorporated, whereas the SERS spectra of biomolecules may be less distinct from one another. The use of data processing methods including chemometrics and machine learning can also improve the output of SERS analysis by increasing the accuracy of data interpretation. However, the preferred method is highly dependent on the end goal of the test, the desired application, the target biomolecule, and the biological matrix that will be sampled.

SERS-based detection of biomolecules has been well studied over the years and the technique offers excellent capabilities in terms of sensitivity, specificity and multiplexing. Despite the strengths of SERS for biomolecular detection, the technique is not commonly adopted in end-user applications. Considerations such as NP toxicity, laser safety, time, cost, reproducibility, detection in biological matrices, and the adaption of SERS analysis into point of care (POC) tests have been stumbling blocks in the clinical application of SERS. Additionally, it is difficult to convince end-users that SERS is a worthwhile replacement for their current “gold standards”. However, there is a growing need for continuous health monitoring and personalized healthcare due to our aging populations, reduced resources, and strains on health boards. This has resulted in a drive toward POC detection, wearable sensors, and tests that can be simply and quickly carried out in primary care settings or by patients at home. The advantages of SERS are well-suited to these requirements and research has begun to highlight this by demonstrating the use of the technique for the rapid and sensitive analysis of biological samples. Additionally, the development of portable instrumentation has enabled SERS detection to be implemented at the point of use and machine learning methods have emerged to enable improved data analysis and therefore more accurate diagnosis in a rapid time frame. This perspective will discuss the potential of SERS to be integrated into true POC applications for real-time human health monitoring, with a particular focus on minimally invasive approaches.

## Noninvasive Point of Care Testing

### SERS-Based Lateral Flow Immunoassays

**Figure 1:**
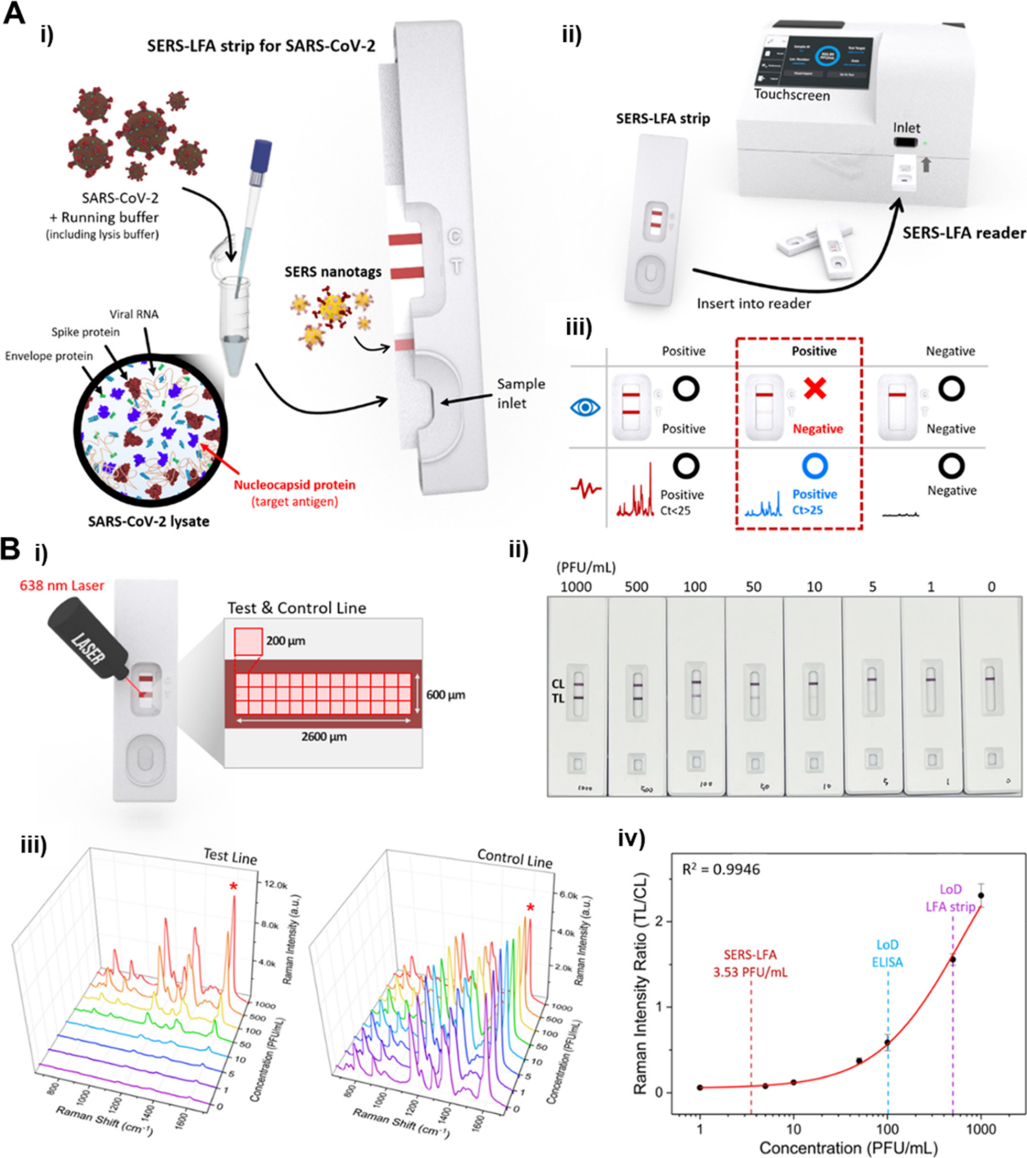
A. (i) Assay concept for the SERS-LFIA for SARS-CoV-2, (ii) analysis of the SERS-LFIA strip on a portable lateral flow Raman reader, (iii) example of a positive SERS result that would have yielded a negative result using a commercial LFIA test. B. (i) Photo of a SERS-LFIA strip for 1000 PFU/mL SARS-CoV-2 and the pixel-to-pixel detection process for the test and control lines, (ii) photo of the SERS-LFIA strips for 0–1000 PFU/mL SARS-CoV-2 concentration range, with test line only visible to 50 PFU/mL, (iii) average SERS spectra of the test and control lines based on the intensity of the peak at 1602 cm^–1^, (iv) calibration curve for the SERS-LFIA based on the Raman peak intensity ratios (test line (TL)/control line (CL)) at 1602 cm^–1^ for 1–1000 PFU/mL SARS-CoV-2.^20^ Adapted with permission from ref (20). Copyright 2022 American Chemical Society.

The potential of SERS-based POC assays has been well documented and various platforms have been investigated for the detection and diagnosis of many different diseases.^16^ In recent years, the combination of SERS with lateral flow immunoassays (LFIAs) has gained a huge amount of attention by enabling low-cost user-friendly LFIAs to become quantitative and more sensitive, with the capability to detect multiple biomarkers simultaneously.^17^ SERS-based LFIAs improve on SERS-immunoassays by carrying out the detection on an inexpensive paper-based strip in under 20 min. The test area can then be analyzed with a Raman spectrometer and the intensity of the signal related to the concentration of biomolecule present. In keeping with the POC nature of the LFIA, the SERS analysis can be carried out on a portable Raman spectrometer, enabling the implementation of the tests in a variety of POC applications for rapid, quantitative detection of biomarkers.^18^ The timing of the coronavirus 2 (SARS-CoV-2) pandemic also helped to highlight the potential of SERS-LFIAs as the need for such a rapid and sensitive detection platform was so vast. Commercial SARS-CoV-2 tests were quick and convenient but due to limitations in sensitivity, false negative results were often obtained in the presence of symptoms or for nondetectable but contagious levels of the virus. SERS-based LFIAs overcame this issue by enabling earlier detection of SARS-CoV-2, as well as the ability to distinguish between SARS-CoV-2 and influenza A.^19^ The SERS-based LFIA for SARS-CoV-2 was combined with a portable lateral flow Raman reader for the on-site detection of SARS-CoV-2 and out of 49 positive tests only 2 false negatives occurred, in comparison to the 21 false negatives obtained using the commercial, visual based lateral flow test.^20^ The concept of the SARS-CoV-2 SERS-LFIA and the results obtained using the lateral flow reader are illustrated in Figure 1. This clearly highlighted the potential of SERS-LFIAs for improved sensitivity and the benefits of this when rapid and accurate detection is required, for example to reduce the spread of infection. It also demonstrates that SERS-LFIAs are ready to be implemented into clinical settings and could offer improvements in diagnostics by increasing sensitivity, enabling multiplexing and allowing quantitative analysis of LFIAs. Atta et al. used gold nanocrowns, gold shells decorated with external nanospheres, to produce strong colorimetric and SERS signals in a dual-mode SERS-LFIA.^21^ They applied the assay to enable the ultrasensitive detection of the spike 1 (S1) protein of SARS-CoV-2 and obtained a limit of detection (LOD) of 91 pg/mL for colorimetric detection, with an improvement of 3 orders of magnitude for the SERS detection (57 fg/mL). They also demonstrated the use of the assay for spiked saliva samples without pretreatment, with a detection limit of 40 fg/mL using the SERS-LFIA. A SERS-based LFIA has also been applied for the quantification of biomarkers in whole blood. Liu et al. used magnetic nanotags for SERS enhancement and to enable concentration of biomarkers from unprocessed blood samples.^22^ This assay was applied for the quantitative detection of serum amyloid A (SAA) and C-reactive protein (CRP) on a single lateral flow strip, with detection limits of 0.1 and 0.01 ng/mL, respectively.

There is a push to increase the sensitivity of SERS-LFIA by incorporating “brighter” nanoparticles. For example, gold nanostars, one of the most efficient plasmonic nanomaterials for optical sensing using SERS, has been applied for the detection of chloramphenicol for food safety and human health, with an ultralow detection limit of 10 pg/mL being achieved.^23^ To further increase the SERS intensity, gold nanostars coated in silver have been used for the detection of influenza A.^24^ The strong electromagnetic field generated at the tips of the nanostars and the high extinction coefficient and refractive index of silver produced a strong SERS signal when functionalized with 4-mercaptobenzoic acid and when applied in the SERS-LFIA, influenza A was detected with a LOD of 8 pg/mL. Other nanoparticles used include gold nanocrowns,^21^ gold–silver alloy hollow gold nanoshells^25^ and nonspherical gap enhanced Raman tags.^26^ Although these nanomaterials produce very low limits of detection, the uniformity is often compromised due to their complex shapes and sizes. This can lead to variation in SERS signal and performance on the SERS-LFIA. The synthesis methods are also more complex, again leading to more variation. If SERS-LFIA using these nanomaterials are to be considered for use in clinical applications, standardized, large scale synthesis methods, with many quality control steps will need to be implemented to ensure that every batch of nanomaterials has the same performance as previous batches.

Another consideration when using SERS-LFIA is the pairing of the LFIA strip to the Raman spectrometer. To be used at the POC, a portable Raman spectrometer that is safe to use in clinical environments is needed. To be safe, the laser must be enclosed and operated at low laser powers. Therefore, when performing initial experiments, they should be carried out at the low laser power to ensure that the test sensitivity is not affected. The coupling of the lateral flow strip to a portable spectrometer should also be robust, and the correct focal distance maintained throughout analysis to ensure little variation between measurements. By taking the nanoparticle synthesis and coupling to portable spectrometers into consideration, SERS-LFIA should be viewed as a gold standard POC technique.

### Microfluidic Devices with SERS Detection

Another attractive area that SERS has been paired with that could be used in noninvasive POC applications, is microfluidics. Microfluidic devices, also known as lab-on-chips, are instruments that are designed to handle low volumes of fluids using channels that can be precisely controlled. They can carry out specific tasks including sample pretreatment, separation, dilution, mixing, chemical reaction, detection, and product extraction.^27^ Microfluidic platforms are extremely attractive for POC applications when designed to perform immunoassays as they can sensitively detect clinically relevant concentrations of different biomarkers using a device that is small, uses low sample volumes, has low associated reagent costs, is reusable and produces rapid results.^28^

When pairing SERS with microfluidic devices, the SERS substrate can either be injected into the chip as a SERS nanotag that is designed to bind to an analyte of interest and then immobilized onto a detection zone, or integrated into the chip where it can enhance the Raman scattering of an analyte of interest that has been directed on to it. Regardless of how the SERS substrate is incorporated into the device, it is important to consider the challenges associated with them, which is mainly poor reproducibility in the SERS signal. To overcome the lack of reproducibility, Choi et al. used an internal standard (IS) approach when integrating SERS nanotags into a microfluidic device for the automated immunoassay detection of antigen fraction 1 (F1) in *Yersinia pestis*.^29^ The IS inclusion accounted for variation of the SERS substrate in a droplet, formed via the injection of an oil in a droplet generation compartment, thus increasing the reproducibility and improving the quantitative detection. In a similar approach, SARS-CoV-2 was detected using a SERS-based microdroplet sensor.^30^ The authors compared the microfluidic approach that analyzed 140 droplets traveling through the microfluidic channel and focal volume of the laser over 15 s, to a 5-point scan of the supernatant collected after magnetic separation via a conventional SERS-based magnetic bead assay in a microtube. Using the microfluidic channel the limit of detection improved from 36 to 0.22 PFU/mL and the coefficient of variation from 21.2% to 1.79%, giving compelling evidence that SERS combined with microfluidics can be reproducible. Furthermore, when clinical nasopharyngeal aspirate samples were evaluated, the results agreed well with reverse transcription-polymerase chain reaction results. Although not demonstrated, they envisaged that it could be easily integrated with a portable Raman spectrometer and used as a POC diagnostic platform. Another example of a SERS-based microfluidic with excellent reproducibility has been reported by Lu et al.^31^ In their platform, a unique nanocone array with nanoscale wrinkles acted as the solid capture plate and SERS substrate for an immunoassay designed to detect dual prostate cancer markers. The nanocone array covered with gold film provided a large surface area for aptamer conjugation allowing sandwich immunocomplexes to form and when analyzed with SERS could detect prostate-specific antigen and thrombin with detection limits of 0.01 ng/mL and 0.01 nM. The substrate also produced a relative standard deviation of 7.4%, indicating good uniformity and showing that SERS-microfluidics do indeed have good reproducibility. Wu et al. demonstrated the capabilities of SERS-microfluidic platforms for POC testing using a hand-powered microfluidic approach for the SERS detection of circulating tumor DNA in whole blood.^32^ In their method, the preprocessing of the blood was carried out on-chip and the SERS-based amplification-free detection of DNA mutations was achieved in 35 min.

In these examples, the SERS analysis was carried out on large Raman microscope systems, with scope to transfer the analysis to portable Raman spectrometers. However, there are only a few examples that have actually demonstrated the pairing of microfluidic devices with portable detection. Mabbott et al. reported the detection of cardiovascular disease (CVD) biomarker miR-29A using portable SERS combined with paper-based microfluidics.^33^ In this example, the three-dimensional paper-based microfluidic device was designed to detect mir-29a using a split hybridization assay and the detection zone was interrogated using a portable Raman spectrometer, with a 3D printed interface to pair the device with the spectrometer to increase the reproducibility of measurements. The authors suggest that when paired with fingerprick blood samples, the quantitative paper-based assay should be used for POC applications for CVD diagnosis. Although excellent sensitivity has been achieved, to fully harness the portable nature of microfluidics and their combination with SERS for sensitive and rapid biomarker detection at the POC, the SERS community must carry out further investigations to push this platform as a gold standard approach with POC detection. This can be achieved by testing the sensitivity and specificity using clinical samples and working on the interface between the microfluidic device and portable spectrometers.

For detection and diagnosis of many conditions, blood testing is the current gold standard. However, many biomarkers can also be detected in alternative biofluids such as urine, which is easier to collect and can be sampled when needed.^24,25^ This is advantageous in the move toward POC testing, where minimally invasive sampling is desirable. Ultimately, in POC testing, the most suitable biofluid and test platform are dependent on the specific requirements and end goal of the application. The main considerations are the sensitivity, speed and cost of the test and the presence of relevant biomarkers in the target biofluid. The simplicity of carrying out the test and interpreting data are also vital considerations but are dependent on where the test will be taken and who will run it. For example, tests carried out in hospitals, GP clinics or at home each have different requirements in their ease of use, although user-friendliness is always desirable. In current times, efforts are being made to reduce the need for regular clinic visits and the public have an increased awareness and desire for personal health monitoring. This has led to the development of wearable sensors to enable *in situ* health monitoring and personal tracking. Examples of this include the widespread use of smartwatches and rings that can track activity, resting heartbeat and measure blood oxygen levels. However, as the population strives to learn more about their health, the ability to obtain more detailed information through personal devices is desirable, especially for the personal monitoring of chronic conditions such as diabetes. This can be achieved by incorporating SERS detection with wearable sensors for real-time sensitive monitoring of health indicators.

## Wearable SERS Sensors

The ability to obtain *in situ* information about health status at the molecular level is highly beneficial for human health monitoring. This has resulted in a large volume of research on the development of wearable sensors, where direct information can be obtained from biofluids, such as tears, sweat, saliva and interstitial fluid.^34^ This is even less invasive than, for example, blood sampling, and is an appealing alternative for personal health monitoring. Wearable sensors can be combined with portable instrumentation, sensitive detection methods, and sophisticated hardware, to enable true POC analysis and potential for personalized healthcare. Various outputs have been incorporated for wearable sensing, such as colorimetric detection, fluorescence and electrochemistry.^35−37^ Optical methods like colorimetry and fluorescence are straightforward and can be combined with smartphones to yield simple, user-friendly devices;^38^ however, they tend to lack capabilities for continuous monitoring and do not provide specific molecular information. Electrochemical devices have been developed for continuous monitoring, but these methods often require complex electrode design, can suffer from interference, and limited information is obtained from samples. SERS is a suitable alternative that can be combined with wearable sensors to obtain detailed information from biofluids in real-time without the need for labeling.

### Design of SERS Substrates for Wearable Sensing

**Figure 2:**
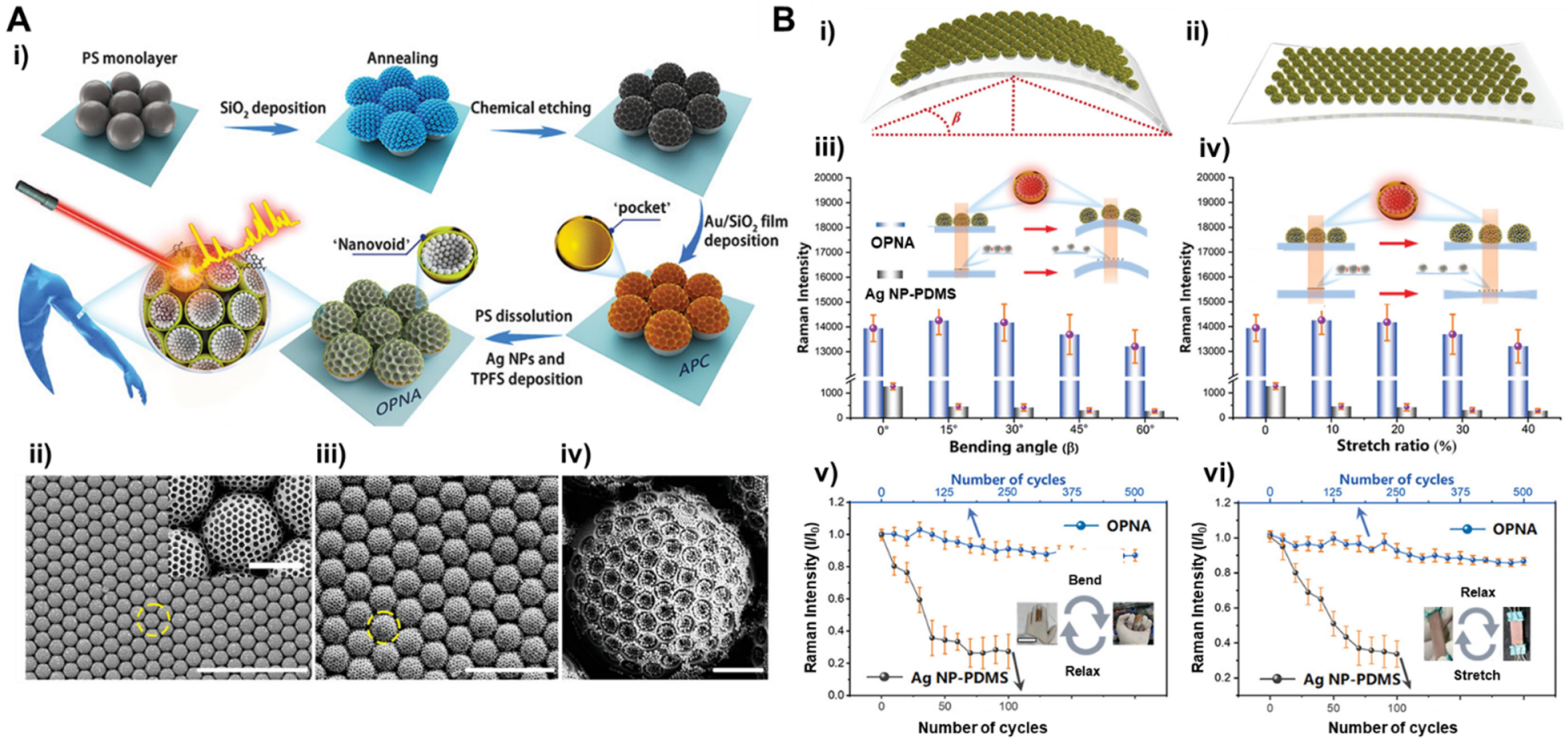
A. (i) Preparation process of omnidirectional plasmonic nanovoids array (OPNA) substrate. SEM images of (ii) artificial plasmonic-compound (APC) and (iii) OPNA substrate, (iv) the enlarged image in the yellow circle in (iii). Scale bars: (ii) 25 μm, inset in (ii) is 2, (iii) 10, and (iv) 2 μm. B. Sketches of an OPNA sensor under (i) bend and (ii) stretch. SERS responses of an OPNA sensor and Ag NP-PDMS under (iii) bend and (iv) stretch, the insets in (iii) and (iv) are the changed “hotspots” in OPNA and Ag NP-PDMS under deformations, respectively. SERS characteristics of OPNA and Ag NP-PDMS after the cycles of (v) bending and (vi) stretching test. The error bars in (iii)–(iv) indicate the standard deviation of signal intensity during four measurements. Scale bars of insets in (v) and (vi) are 1 cm.^45^ Adapted with permission from ref (45). Copyright 2022 Wiley-VCH GmbH.

Important considerations for the application of wearable sensors are sensitivity, to enable detection of analytes at low concentration directly from the biofluid; flexibility, so that the signal is not affected by movement, such as bending and stretching; durability, so the devices are not damaged during wear; stability, to ensure consistent performance over time; and biocompatibility, to avoid irritation when worn. To this end, research on the design of SERS-based wearable sensors is largely focused on the fabrication of scalable, reproducible, low-cost and robust substrates that enable simple sampling and sensitive detection. This involves the incorporation of strongly enhancing nanomaterials into flexible and durable platforms, such that molecular information can be obtained from the SERS spectra of analytes, even at low concentration. Flexible and bend-insensitive SERS substrates have been developed to ensure stable and homogeneous SERS signals on curved surfaces over significant time periods, and during the movement that would be experienced upon wearing.^39−41^ Nanofibers have been used as low-cost, highly scalable substrates that can be simply manufactured by electrospinning polymers and coating with Au to form SERS-active substrates for wearable sensing.^42,43^ Self-adhesive and reusable substrates have been designed to ensure comfort and stability in performance for wearable sensors.^44^ Efforts have also been made to design substrates where the SERS enhancement is not affected by the position or the angle of incident light. Inspired by the structure of the Xenos Peckii eye, which has a wide-angle detection ability, Zhu et al. prepared an omnidirectional plasmonic nanovoids array (OPNA) by assembling a monolayer of Ag NPs onto an artificial plasmonic-compound eye (APC) (Figure 2A).^45^ The APC is an interconnected frame with omnidirectional “pockets” for enhancement of hotspots, which also protects the hotspots against mechanical deformation. Sensitive detection was achieved using the substrate, with a limit of detection (LOD) of 10^–16^ M for rhodamine 6G (R6G). To demonstrate the practicability of the sensor, a simulated on-body test was performed using a human sweat mimic and sensor deformation to account for changes during exercise. The authors selected dopamine as the analytical model to study as its quantitation can help understand neurological disease or emotional activities. The results demonstrated the sensor could accumulate 2 μL of sweat in the testing zone and when analyzed with SERS, could detect 1 pM of dopamine. The SERS signal also remained stable when the substrate was bent and stretched, or when rubbed to test wear resistance. When compared to Ag NPs deposited on flexible polydimethylsiloxane (PDMS), the OPNA substrate exhibited significantly better stability during bending and stretching, with little defects and variation of signal (Figure 2B). Lv et al. used another nature-inspired approach and developed a wearable SERS sensor based on a bionic sea urchin-cavity (BSC) structure.^46^ The BSC structure has high rotational symmetry that enables the sensor to make full use of incident light regardless of reverse excitation, tilting and bending, which is ideal for wearable sensors. Copper nanowires (Cu NWs) were incorporated for efficient adsorption of analytes and were coated in Ag to yield high intensity SERS hotspots for signal enhancement. The BSC structure has a high electromagnetic field that is less affected by changes in angle of incident light, such that the SERS signal was stable when the substrate was bent or when a nonvertical laser excitation was employed. The SERS-active surface gave good signal enhancement, with LODs of 10^–15^ M for R6G, 10^–10^ M for urea and 10^–6^ M for lactic acid. Furthermore, when it was applied as a wearable sensor, it was able to detect slight changes in urea concentration on human skin when in a resting state. The substrate was also tested for the detection of volatile organic compounds (VOCs) and a Raman spectrum of acetone was obtained from gas volatilized from a 30 mmol/L aqueous solution, which is similar to the acetone concentration in diabetic blood. The substrate therefore demonstrated potential as a wearable sensor for on-skin detection of metabolites, as well as for breath analysis.

### Transfer of Biofluids to SERS Substrates

Efficient transfer of the analytes to the SERS substrate is essential for detection and various approaches have been investigated. Paper microfluidics are a cost-effective and disposable option that enable simple capture of biofluids through capillary action and have a high surface area so that a high density of NPs can be deposited for SERS enhancement.^47,48^ Paper-based devices are also advantageous for continuous monitoring where the flow of the analyte through the microfluidic device allows changing analyte concentration to be quantified, either by continuously scanning a single sensor or by detection at multiple sensors along the microfluidic channel. Paper-based devices can also be used to monitor additional properties, such as sample volume and pH, by measuring the distance moved by the biofluid or incorporating pH indicators.^49^ Li et al. designed a flexible plasmonic paper-based microfluidic device with expandable channels that could be used to control the flow rate of biological fluids.^48^ They demonstrated the flexibility, strength and biocompatibility of the device as a wearable sensor and showed that it could be used to detect uric acid (UA) from human sweat *in situ* using a portable Raman spectrometer, with a laser blocking layer incorporated to prevent skin damage during measurements. They also demonstrated the feasibility of the approach for continuous monitoring by sequentially adding varying UA concentrations and showing that the SERS intensity increased and decreased with UA concentration. Silk fibroin films (SFFs) can also be used for biofluid extraction as they are flexible, highly absorbing and biocompatible.^50−52^ The SFF can also act as a filter to allow absorption of the target analytes while trapping larger molecules to avoid interference. Koh et al. demonstrated this by analyzing Raman reporters of varying molecular weight and showing that a SERS signal was obtained for the smaller molecules that could pass through the SFF layer, while larger molecules were trapped, and no SERS signal was observed.^50^ Lee et al. highlighted this benefit for separating small molecule analytes, such as glucose, from the proteins present in biofluids that could potentially interfere with analyte detection.^51^ Fabric sensors have also been developed where hydrophobic and hydrophilic layers are used to efficiently transfer and collect the sample.^53,54^ NPs can then be embedded into the fibers for SERS enhancement or SERS nanotags can be incorporated for recognition and detection of target analytes. Hydrogels have also been utilized for their strength, biocompatibility, porosity and flexibility. Wang et al. used a sulfonated cellulose nanocomposite hydrogel (S-CNF-Ag NPs/PAA), where they incorporated sulfonated cellulose nanofibers (S-CNFs) into their Ag NP synthesis, then UV cross-linked with acrylic acid. S-CNFs are strong and renewable biomaterials with hydroxyl and sulfonic acid groups on the surface that can help stabilize the Ag NPs, while improving mechanical toughness and adhesion of the hydrogel. This results in a biocompatible and porous hydrogel that can hydrogen bond with the skin and effectively absorb biofluids, with minimal loss by evaporation due to the cross-linked network. The hydrogel enables the effective trapping of the analytes, which can be detected due to the SERS enhancement provided by the Ag NPs. Methods have also been investigated to induce the production and extraction of biofluids. Wang et al. developed a plasmonic electronic device with an electronic sweat extraction element and a plasmonic component for SERS sensing.^55^ They used two flexible electrodes in a “yin-yang” design, with a thin hydrogel film containing a sweat extracting drug. This induced sweat extraction using an iontophoresis process and a silver nanocube (Ag NC) superlattice film was inserted through a hole in the hydrogel as the SERS sensing component. Both components were bonded to a thin polymer film for protection and skin adhesion and *in vivo* tests were carried out on human volunteers. The sweat extraction process was tested by measuring skin moisture content after stimulation. A significant increase in moisture was observed and could be controlled by altering iontophoresis time.

### Applications of Wearable SERS Sensors

**Figure 3:**
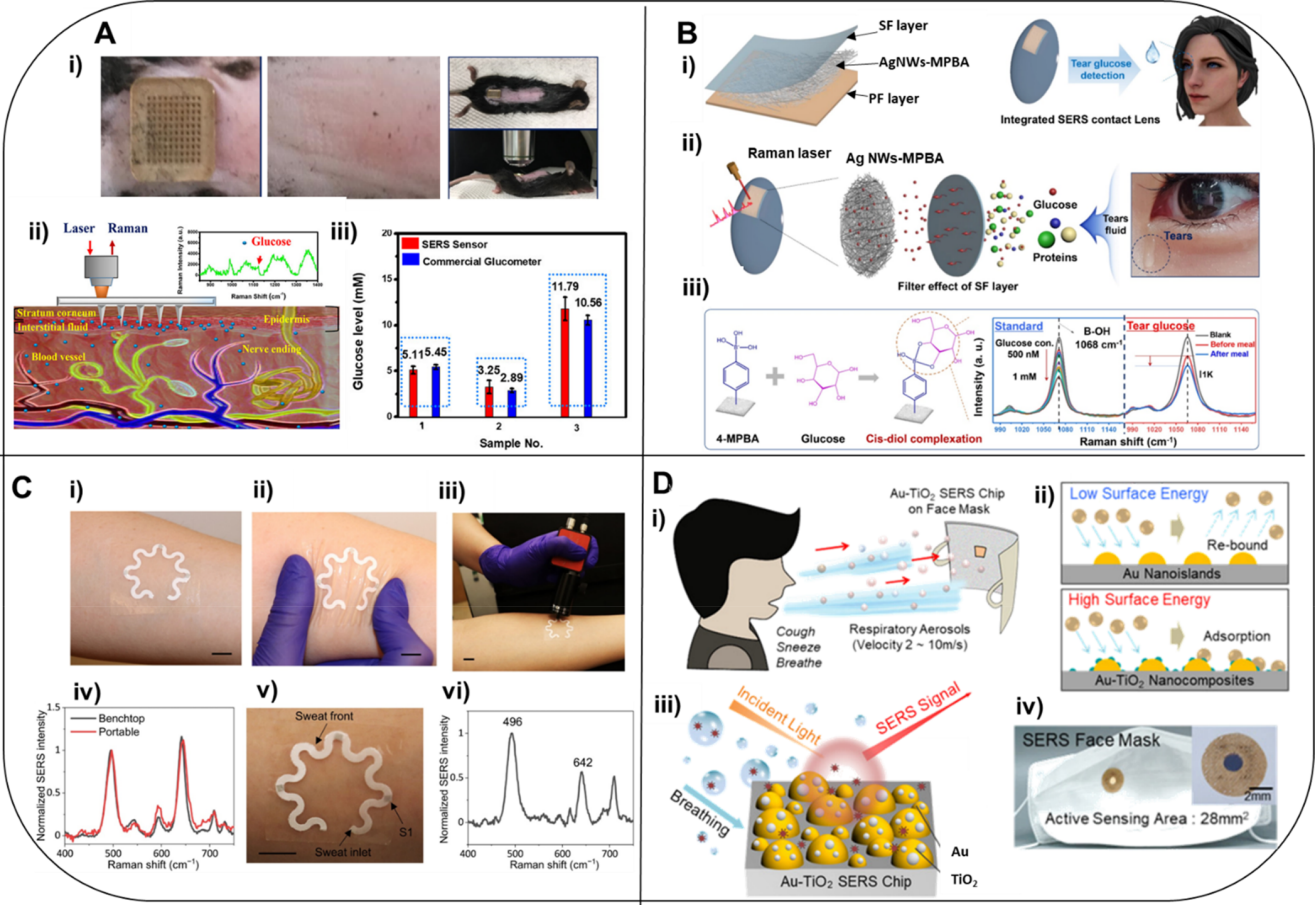
Applications of wearable SERS sensors. A. Low-cost poly(methyl methacrylate) microneedle (PMMA MN) array for *in vivo* glucose measurement from interstitial fluid in a mouse model.^59^ (i) Photo of the F-PMMA MN array on the skin on the back of the mouse (left), the mouse skin 10 min after array removal (middle), and photos of sensor on the mouse and spectral collection setup (right); (ii) schematic of *in vivo* transdermal detection of glucose; (iii) glucose levels measured using SERS glucose biosensor (red) and a commercial glucometer (blue). Adapted with permission from ref (59). Copyright 2020 American Chemical Society. B. SERS contact lens material (SERS-LM) for analysis of glucose in tears.^51^ (i) SERS-LM structure (silk fibroin (SF) layer for analyte absorption and filtering, Ag NWs-MPBA for SERS, and protective film (PF) layer); (ii) selective glucose detection mechanism using SERS-LM; (iii) chemical selectivity of 4-mercaptophenylboronic acid (4-MPBA) for glucose, and the representative change in Raman spectrum after reaction with glucose at various concentrations (left), and human tear glucose before and after a meal (right). Adapted with permission from ref (51). Copyright 2021 Elsevier B.V. C. Skin sensor for sweat analysis.^47^ (i) Device conformally laminated on the forearm of a human subject, (ii) under deformation, and (iii) a portable Raman spectrometer with a flexible fiber probe for spectra collection. (iv) Comparison of SERS spectra collected with benchtop and portable spectrometers. (v) Photograph of the device after a healthy human subject wore it and exercised for 20 min. (vi) SERS spectrum of the sweat collected from the sensor S1 in (v). Scale bars, 1 cm. Adapted with permission from ref (47). Copyright 2022, The American Association for the Advancement of Science. D. SERS face mask as a wearable breath sensor.^69^ (i) Schematic illustration of SARS-CoV-2 detection from respiratory breath aerosols using the Au-TiO_2_ SERS chip on a face mask. (ii) Aerodynamic behavior of aerosols impacting on a solid substrate with different surface energies. Au-TiO_2_ nanoislands with high surface energies efficiently adsorb the low-volume and high-velocity respiratory aerosols. (iii) SERS detection of SARS-CoV-2 aerosols. (iv) Photograph of the SERS face mask as an application example. Reprinted (adapted) with permission under a Creative Commons Attributions 4.0 International License from Hwang, C. S. H.; Lee, S.; Lee, S.; Kim, H.; Kang, T.; Lee, D.; Jeong, K.-H., Highly Adsorptive Au-TiO2 Nanocomposites for the SERS Face Mask Allow the Machine-Learning-Based Quantitative Assay of SARS-CoV-2 in Artificial Breath Aerosols. *ACS Appl. Mater. Interfaces***2022**, 14, 54550–54557. Copyright 2022 The Authors.

Ultimately, the most effective SERS substrates and efficient methods of transfer are dependent on where the sensor will be worn and what biofluid will be sampled. As outlined, many approaches have been explored and each have benefits and disadvantages depending on the target application. Figure 3 highlights four types of wearable SERS sensor that have been applied for different sampling approaches, for the analysis of interstitial fluid (ISF), tears, sweat and breath.

ISF is the fluid surrounding cells, which is similar to blood plasma and has almost the same biomarker content as blood.^56^ As opposed to drawing blood or using a finger-prick approach, ISF can be painlessly accessed through the dermal layer of the skin using minimally invasive sampling, making it a more desirable biofluid for continuous monitoring. ISF has been investigated for diagnostic applications and, in particular, for glucose monitoring in diabetes management.^57,58^ Wearable SERS sensors for analyzing ISF have been developed in the form of microneedle arrays that can be applied to the skin to enable *in situ* intradermal measurements. An example of this is shown in Figure 3A that demonstrates how a SERS microneedle biosensor was used for *in vivo* glucose detection from mouse ISF.^59^ The microneedle array was fabricated using a commercially available polymer, poly(methyl methacrylate) (PMMA), which was then coated with Ag NPs for SERS enhancement and 1-decanethiol (1-DT) for glucose capture. PMMA is a low-cost material that is biocompatible and mechanically strong enough to penetrate skin, but causes less skin damage than alternative materials, such as stainless steel. As shown in Figure 3A (i), the microneedle was minimally invasive, as there was little skin damage observed 10 min after removal of the microneedle array. The microneedles allow sufficient skin penetration to sample ISF but without reaching the dermis layer that contains blood vessels and nerve endings (Figure 3A (ii)). In addition, the PMMA has high light transmittance, such that SERS measurements can be carried out *in situ*. The results of the quantification of glucose by SERS using the PMMA microneedle array were comparable to a commercially available glucometer, indicating the applicability of this device for patient glucose monitoring. SERS microneedles have also been used for *in vivo* drug detection. Li et al. adsorbed Au NPs onto a PMMA microneedle array then grew Ag NPs on the surface of the Au to form silver–gold (Au@Ag) core–satellite NPs for improved SERS enhancement.^60^ The Au@Ag microneedles were then coated with a protective hydrogel layer, which also helped to extract ISF and promote the adsorption of drug molecules onto the SERS substrate. This enabled the real-time detection of trace levels of drugs in ISF and a comparison of the drug concentration in ISF versus blood, which was shown to be drug dependent. In addition to label-free sensing, SERS microneedles have also been labeled with Raman reporters to monitor properties such as pH, redox potential and levels of reactive oxygen species (ROS),^61^ or functionalized with SERS tags for detection of biomarkers.^62^ This is further demonstration of the versatility of SERS sensing and the ability to alter the detection strategy to suit the desired application, thus highlighting the applicability of the technique for monitoring human health.

Despite the advantages of ISF for minimally invasive sensing, microneedles suffer from low sample volume and some analytes, such as glucose, have lower levels in ISF than in blood. An alternative approach to wearable glucose sensing is to use a plasmonic contact lens for monitoring glucose levels in tears (Figure 3B).^51^ As the Raman signal of glucose is weak, glucose sensing can be better achieved by monitoring the glucose-induced shift in the spectrum of 4-mercaptophenylboronic acid (4-MPBA).^63^ Complexation between glucose and 4-MPBA results in suppression of the “breathing” mode of 4-MPBA at 1071 cm^–1^ and an increase in the constrained bending mode at 1084 cm^–1^, causing a shift in the dominant peak. Yang et al. used this spectral shift for the detection of glucose in the range of 0.1–30 mM.^63^ As no Raman peaks for glucose were observed at 10 mM, this was a significantly improved method over direct glucose detection. Additionally, analyzing the shift in Raman bands, rather than intensity, allows it to be independent of the substrate and other experimental factors. This work also demonstrated the capability of the method in wearable sensors by implanting the substrates in *ex vivo* rabbit eyes and measuring glucose concentrations, which were within 0.5 mM of a commercial spectrometer. Lee et al. developed a SERS contact lens material (SERS-LM) using a layered approach, where they used a SFF for analyte absorption and filtration, a layer of silver nanowires (Ag NWs) coated with 4-MPBA for SERS sensing, and a protective film (PF) to prevent contamination (Figure 3B (i) and (ii)).^51^ This was used to measure glucose concentration by monitoring the decrease in the Raman band of 4-MPBA at 1068 cm^–1^ on binding with glucose. Glucose was detected in the range of 500 nM to 1 mM, with a LOD of 211 nM being achieved (Figure 3B (iii)). To demonstrate the practical use of the device, human tears were analyzed before and after a meal and similar trends were observed to the glucose levels in blood. Although the device was not applied *in vivo*, it demonstrated the potential of tear glucose monitoring by integration into disposable contact lenses.

Various wearable SERS sensor platforms have been described and their applicability has been demonstrated for several applications in the analysis of different biofluids. However, the majority of wearable SERS sensors have been developed for the analysis of sweat. Sweat is very easy to collect, less invasive than other biofluids, and contains metabolites and electrolytes that can reflect health conditions (e.g., glucose, urea, uric acid). Other properties of sweat, such as pH, can also be analyzed to monitor health. Drug concentration in sweat can also be measured using wearable SERS sensors, which could be useful for drug abuse testing, antidoping control, monitoring medicinal efficacy and health analysis.^50,55,64^ Sweat sensors can also be worn on body parts, such as arms or forehead, that are accessible for detection and can be easily analyzed *in situ* to enable continuous monitoring. An example of a wearable sweat sensor is shown in Figure 3C. Mogera et al. developed a soft, flexible and stretchable paper-based microfluidic device for the continuous and simultaneous quantification of sweat loss, sweat rate and concentration of metabolites in sweat.^47^ The microfluidic device had a serpentine design that was flexible and stretchable to accommodate skin deformation (Figure 3C (i) and (ii)). As shown in Figure 3C (iii), a hand-held Raman spectrometer was used to analyze the sensor *in situ*. A thin layer of carbon tape was placed between the device and the adhesive to protect the skin from laser-induced damage, and plasmonic sensors (gold nanorods (AuNRs) embedded in chromatography paper) were immobilized at different points along the microfluidic channel to allow the detection and quantification of analytes at different time points using SERS. In comparison to a benchtop instrument, there was no loss in signal when using the portable, hand-held spectrometer (Figure 3C (iv)). The movement of the liquid along the microfluidic device could be clearly observed so the sweat volume and rate could be determined (Figure 3C (v)). The device was also applied for the detection of uric acid, which is commonly analyzed in serum for nutritional and metabolic management and is associated with health conditions such as cardiovascular disease, renal disease and gout.^49,65^ Uric acid was measured in buffer and artificial sweat and could be detected to 1 μM and without interference from other molecules. They also demonstrated the capability of the device for continuous monitoring by sequentially adding different concentrations of UA and showing the corresponding change in SERS signal intensity with the rapidly changing UA concentration. This was carried out using two different approaches. The first was by continuously scanning one sensor in the microfluidic channel to quantify the changing concentration of UA when sequentially adding increasing and decreasing concentrations. The ratiometric SERS intensity at the sensor increased and decreased with changing UA concentration, indicating the potential applicability of the device for continuous monitoring. In the second mode, multiple sensors were spatially distributed along the microfluidic channel and the samples were scanned at the end point. In this method, the SERS intensity at each sensor varied corresponding to the sequentially changing UA concentration, demonstrating the capability of the device to measure changing analyte concentration over time. A healthy human volunteer wore the sensor for 20 min while running and experienced no skin irritation. 13.7 μL of sweat was collected and the concentration of uric acid in the sample was 28 μM, which is consistent with the levels of a healthy individual.^65^ Chen et al. also used a paper microfluidic sensor for uric acid detection in sweat.^49^ They added a pH indicator to their device and used a phone to determine pH and volume of sample. They also spiked sweat samples with uric acid to stimulate gout and used artificial intelligence (AI) methods (linear discriminant analysis, LDA, partial least-squares, PLS, and artificial neural networks, ANN), to separate the spiked versus nonspiked samples. Wang et al. demonstrated the use of their nanocomposite hydrogel-based sensor to detect urea and uric acid detection over interferents in sweat, with LODs of 63.1 and 3.5 μM, respectively. They confirmed that the analytes were detectable within the range of concentrations normally found in sweat on human skin and that the sensor had antimicrobial properties and good biocompatibility, which are important in the application of wearable sensors.

SERS-based wearable sensors can also be used to monitor the concentration of multiple metabolites and biomolecules simultaneously, which can give more detailed information about physiological state and health conditions.^66,67^ In a recent paper, Atta et al. introduced a simple, wearable SERS sensor and demonstrated its use for the simultaneous detection of multiple analytes from human sweat.^67^ They dropped Au nanostars onto an adhesive tape and established that reasonable SERS enhancement could be attained from the simple substrate with a LOD of 0.01 nM for R6G. LODs were also obtained for three biomarkers, glucose, lactate and urea, which were found to be significantly lower than the clinically relevant concentrations. The sensor was then applied to simultaneously measure the concentration of the three analytes in human sweat during sitting, walking and running. This demonstrates the capabilities of wearable SERS for the real-time detection of multiple biomarkers from sweat.

In addition to the direct detection of metabolites from sweat, sweat pH can also be used to check for dehydration and to identify skin disorders, including acne and dermatitis. It can also be used as an indicator of hypoglycaemia, which needs medical intervention, in diabetes. Wang et al. used their wearable device to monitor sweat pH by modifying the sensor with a pH-sensitive Raman reporter, 4-mercaptobenzoic acid (4-MBA).^68^ The sensitivity of pH detection was in the range of human sweat (pH 5.5–7.0) and measured pH values the same as a standard pH meter. Chung et al. formed self-assembled monolayers (SAMs) of 4-mercaptopyridine (4-MPY) and 4-MBA on their nanofiber substrates for pH sensing of sweat.^42^ They suggested using a hybrid approach with both Raman reporters to optimize detection accuracy. They achieved accurate and stable pH sensing over the sweat pH range (pH 4–7) using sample volumes as low as 1 μL, with readings from human sweat samples comparable to those obtained using a pH meter. As described, there are many advantages to analyzing sweat using wearable SERS sensors and a wealth of information can be obtained in real-time using minimally invasive *in situ* detection.

Although less common, breath is another biological sample that can be assessed to monitor metabolic changes that occur in diseases, such as cancer. The SERS breath sensor shown in Figure 3D illustrates an alternative approach to wearable sensing, where a SERS substrate was incorporated into a face mask for the analysis of breath.^69^ The substrate used Au-TiO_2_ nanocomposites to preconcentrate and capture the breath aerosol to enhance detection sensitivity by 47% over Au nanoislands without the TiO_2_. This platform was used for the direct, label-free detection of SARS-CoV-2 in respiratory aerosol using a “breath biopsy”. The SERS face mask was paired with machine learning to enable a quantitative assay direct from breath for 10^1^ - 10^4^ pfu/mL, which was comparable to 19–29 polymerase chain reaction (PCR) cycles from COVID-19 patients. This wearable sensor is an example of how exhaled air can be used to diagnose health conditions in patients using totally noninvasive sampling. The following section will contain further discussion on the analysis of breath for healthcare applications, by exploring the SERS-based detection of volatile organic compounds (VOCs).

Overall, significant advancements in the development of nanomaterials and nanotechnology have enabled the design of sensitive and stable SERS substrates that could potentially be applied for *in situ* health monitoring. Paired with progress in device miniaturization, this makes SERS applicable for personalized healthcare. However, practical considerations in the large-scale fabrication of substrates and in signal stability in the long term continuous analysis of biofluids remain a challenge for wearable sensing. Additionally, spectra can often be complex and therefore data interpretation is challenging. Nonetheless, this is a promising field and continued advances in nanotechnology and data analysis could overcome the challenges.

## Detection of VOCs Using SERS

VOCs are emitted as gas from a variety of different processes. Their detection has been shown for various applications including chemical sensing, homeland security and environmental settings to monitor and increase safety.^70−72^ To detect VOCs, the sample is collected and can be analyzed using photoionization, gas chromatography–mass spectrometry (GC-MS), ion flow tube MS, laser absorption spectrometry and/or infrared spectroscopy.^73,74^ Although these analyses all provide satisfactory results, the platforms are time-consuming, laborious, can have poor sensitivity and require trained personnel. There is therefore a growing need to combine VOC detection with a faster, simpler analysis method, which could also be applied at the POC, and recently SERS has been applied to this application.

To pair with SERS, the VOC of interest must first be adsorbed onto the SERS substrate surface via physical or chemical interactions, however due to the high mobility of gases, this is incredibly difficult. VOCs also suffer from poor adsorption onto SERS substrates as they are small molecules with functional groups that have low or no affinity to the substrates. Furthermore, VOCs have low Raman scattering cross sections, which makes direct label-free analysis difficult to achieve and results in poor sensitivity. To improve, we must use gas enrichment techniques to increase the concentration of VOCs near the SERS substrate, increasing the number of interactions and subsequent adsorption.^75^ To increase the chance of adsorption, the gas can be manipulated via active sampling, which uses an air sampling pump to pull the gaseous sample over the substrate, dynamic headspace sampling, in which an insert gas stream purges VOCs from a sample into a headspace with the VOCs then being transferred to a substrate, or solid phase microextraction (SPME) where a fiber coated with an extraction phase extracts VOCs from a sample before being applied to the substrate.^76−78^ Another method uses indirect tag strategies, which use SERS substrates functionalized with probe molecules that have high cross section and specific recognition elements, to target and capture specific VOCs.^79^ A different approach utilizes metal organic frameworks (MOFs) embedded in the SERS substrate that can concentrate VOCs through their ordered porous structure. This allows them to be nearer the SERS substrate surface and enables them to be detected more readily.^80^ The next section describes how researchers are applying these methods to improve the detection of VOCs via SERS.

VOCs found in foods have been detected using SERS to monitor their quality and safety. Park et al. developed a simple, cost-effective SERS substrate to detect VOCs released from dried teas and live cotton plants.^81^ Their SERS substrate consisted of Ag NPs coated in a thin film of the polymer Tenax-TA. The substrate had a high sensitivity to the VOCs methyl salicylate, phthalate ester and p-cymene, suggesting it could be a useful platform for detecting VOCs with an aromatic group. Taking inspiration from canine animals and their considerable number of olfactory cells, Qu et al. have developed an integrated plasmonic array for the simultaneous detection of multiple food-borne VOCs.^79^ The platform was able to achieve the indirect detection of hydrogen sulfide (H_2_S) using a MOF layer and upon the addition of H_2_S, a new peak at 452 cm^–1^ was observed in the SERS spectrum. Direct detection using a functionalized surface was also investigated. For this, the substrate was functionalized with 4-mercaptobenzoic acid (4-MBA) and the SERS signal intensity of several peaks changed upon the addition of the biogenic amine putrescine, which was used for quantitative analysis. They also utilized an unfunctionalized SERS substrate for label-free direct detection of *P. aeruginosa*. Two new bands at 676 and 2160 cm^–1^ were attributed to the fermentative metabolites of dimethyl sulfide and hydrogen cyanide, indicating *P. aeruginosa* was present. The outputs of this platform significantly improved the sensitivity, reliability, and accuracy for freshness discrimination.

VOCs are also emitted in human breath and the levels can provide an insight into an individual’s physiological and pathophysiological condition.^82^ The major VOCs found in healthy individuals include acetone, ethanol, methanol, isoprene, ammonia, pentane, and many other alcohols, aldehydes, and ketones.^82,83^ Environmental exposure, diet and lifestyle will influence the concentration of VOCs in breath. For example, an increase in acetonitrile and furans will be present if someone smokes.^84^ VOC levels can also be linked to a patient’s health with exhaled ethane and pentane concentrations shown to be elevated in inflammatory disease and increased levels of sulfur containing compounds being linked to liver failure.^85^

Volatile aldehydes are biomarkers of lung cancer, and their detection can be of vital significance in diagnosis and treatment. They are by far the most commonly detected VOC when it comes to SERS-VOC healthcare diagnostic platforms. This is probably due to how easily they can be captured by a SERS substrate functionalized with amines, which undergo a Schiff base reaction with aldehydes in the sample to form imines. However, as the SERS signal of aldehydes are weak, researchers are developing novel SERS substrates to increase the SERS signal and sensitivity. This has been achieved using a dendritic silver nanocrystals substrate functionalized with 4-amino thiophenol (4-ATP) that reacts with benzaldehyde in the sample via the Schiff base reaction. The weak SERS signal was improved by the numerous cavity traps that were present on the dendritic surface, which prolonged the reaction time of gaseous molecules via the “cavity vortex” effect.^86^ This resulted in a significant peak appearing at 1620 cm^–1^, which represented the C=N stretch due to the cross-linking between the −NH_2_ group of the 4-ATP and the −CHO group of benzaldehyde. Overall, the sensor showed good linearity between the range of 2–20 ppm and was selective for aldehydes only. The authors suggest that detecting aldehydes via SERS provided huge potential for screening tests at the initial stages of lung cancer. An alternative SERS substrate for aldehyde detection was designed by Zhao et al., using SERS-active nano traps consisting of plasmonic trimers.^87^ Using 4-ATP, the trimer configuration selectively directed probe molecules to central traps where hotspots were located. This uniform assembly allowed for spatial overlap between molecular adsorption sites and plasmonic hotspots, enhancing the probability that probe molecules experience amplification from the hotspot, improving on a heterogeneous hotspot approach. The platform was used to detect aldehydes from lung tumors using fresh tissue samples. Their findings demonstrate that the approach was sensitive to adenocarcinoma but not squamous carcinoma or benign cancers thereby showing it could differentiate between the subtypes. Using 4-ATP as a probe molecule for aldehyde detection is clearly desirable and it has also been reported to have low limits of detection of aldehyde VOCs with functionalized Au NPs and 3D microneedle arrays coated in Ag NPs.^88,89^

**Figure 4:**
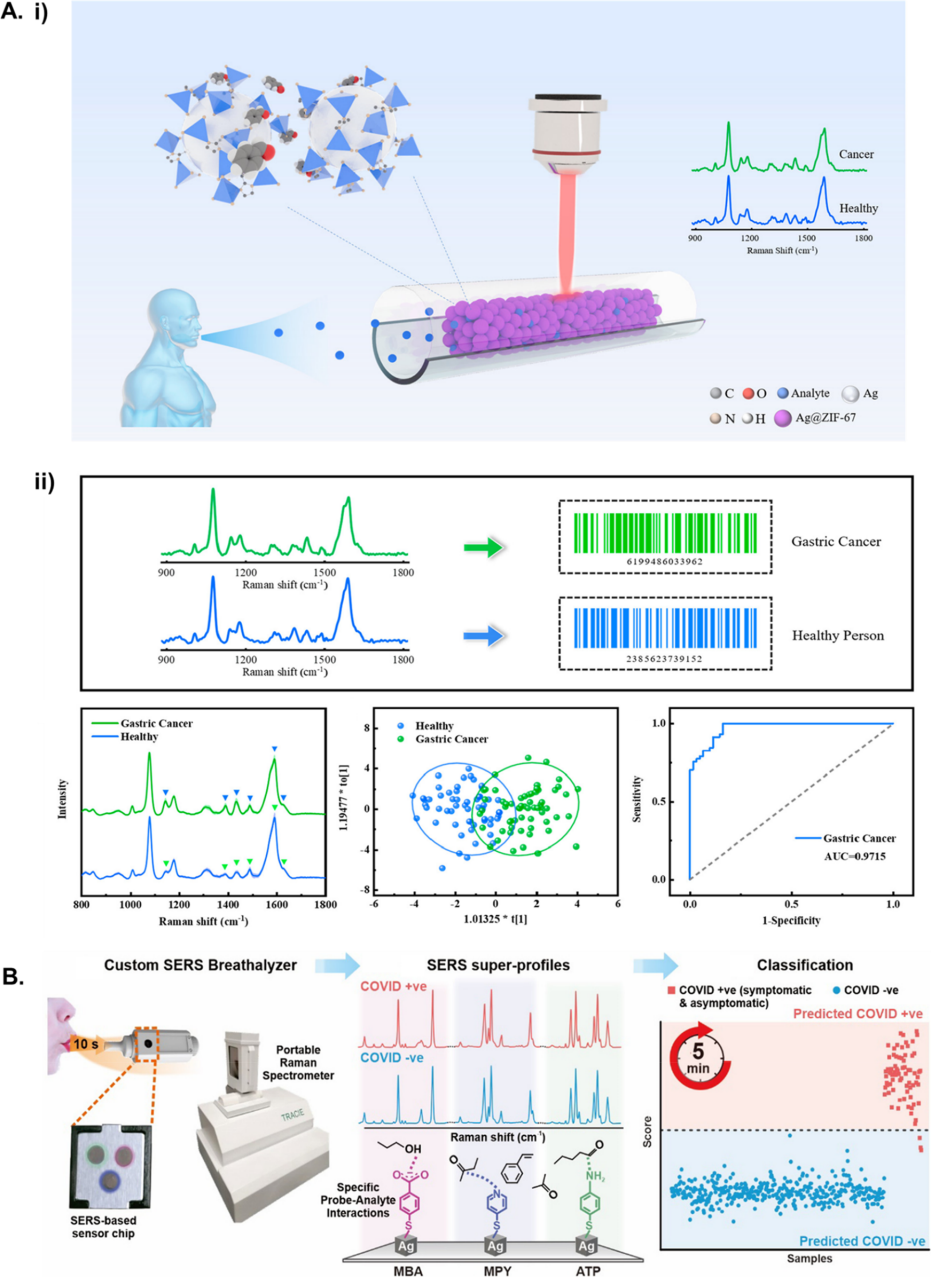
A. (i) Schematic illustration of breath analysis using silver (Ag)@ZIF-67 based tubular SERS sensor for diagnosis of gastric cancer (GC). Breath sample is applied to the sensor that captures aldehydes and ketones. (ii) The SERS spectra from samples of GC patients and heathy volunteers is shown and transformed into barcodes to facilitate practical clinical applications. Orthogonal partial least-squares discriminant analysis (OPLS-DA) plot of SERS spectra shows separation between healthy individuals and GC patients and a ROC curve with an area under the curve value of 0.9715.^92^ Adapted with permission from ref (92). Copyright 2022 American Chemical Society. B. Overview of SERS-based strategy to identify COVID-positive individuals using their VOCs.^98^ First the breath sample is exposed to the sensor which is analyzed using a portable Raman spectrometer. SERS super profiles are obtained based on the binding of the VOCs to the surface functionalized probes MBA, MPY and ATP. The classification using partial least-squares discriminant analysis (PLSDA) score plot shows distinction between breath samples of COVID-positive and COVID-negative individuals. Adapted with permission from ref (98). Copyright 2022 American Chemical Society.

MOFs can also be integrated with 4-ATP functionalized SERS substrates to increase the binding between the aldehydes and substrate surfaces. This has been demonstrated using a smart vapor generation paper-based thin-film microextraction system (VG-PTFM) paired with SERS measurements and was capable of quantifying and detecting benzaldehyde in lung cancer breath samples.^90^ The SERS substrate consisted of core–shell, 4-ATP coated gold nanorods conjugated to quantum dots (GNR-QD)-embedded on a MOF structure. Upon the addition of benzaldehyde, the GNR-QD assemblies were destroyed due to Schiff base reactions between the amine group on the GNR surface and the aldehyde moiety of the benzaldehyde. This produced a characteristic peak at 1620 cm^–1^ that was used for quantification. Lung cancer and healthy subject breath were applied to the device via the VG-PTFM system that was then analyzed using a compact Raman microscope system. The resulting SERS spectra were analyzed using principal component analysis (PCA), which discriminated between the two groups, demonstrating that the platform could effectively identify the different concentrations of aldehyde in lung cancer patients, with similar sensitivity to GC-MS. In a similar approach, Xu et al. detected aldehydes using a TiO_2_ nanochannel membrane that was coated in Au NPs and a gas-trapping MOF layer.^91^ When the gas was passed through the nanochannels, the molecules were trapped in the porous MOF. Again, a Schiff base reaction between 4-ATP and the aldehyde in the sample was used to obtain a robust Raman signal. PCA was used to differentiate what type of gaseous aldehyde was present and the authors suggested this sensor has great promise for lung cancer biomarker screening. An alternative sensor used a glass capillary, acting as a gas flow channel, which was loaded with Ag NPs coated with a uniform MOF (zeolitic imidazole framework-67, ZIF-67) shell functionalized with 4-ATP.^92^ This sensor could produce strong SERS enhancement using the Ag NPs, with the 4-ATP to capture aldehydes and the MOF layer for selective gas enrichment. The platform is shown in Figure 4A. The sensor was used to screen exhaled breath from 57 gastric cancer (GC) patients and 61 healthy individuals, with the SERS results being easily converted into smartphone readable barcodes for facilitating data readout and analysis. Overall, the platform could detect GC with 91.2% and 88.5% sensitivity and specificity, respectively. ZIF-67 has also been applied to concentrate gaseous aldehydes when used to coat Ag NPs, which provided the hotspot and graphitic carbon nitride (g-C_3_N_4_) that formed a membrane to prolong contact time between aldehyde and substrate.^93^

Due to the pore size of MOFs, which are usually microporous, they do have issues with adsorbent blockage. However, this has been overcome by Meng et al., who expanded the pore size by etching MOF structures to form layered double hydroxide (LDH).^94^ They coated silver nanocages in LDH and 4-ATP and applied the sensor for gas adsorption and selective enhancement of benzaldehyde with a limit of detection of 10 ppb using SERS. Furthermore, the sensor was recyclable, with the Schiff base reaction being reversed via hydrolysis.

Aldehydes are well targeted VOC biomarkers, but other VOCs can be detected and related to health concerns. For example, Fu et al. used the MOF MIL-100 (Fe) that comprised of iron clusters and 1,3,5-benzenetricarboxylic acid to target lung cancer VOCs, which included aldehydes but also captured acetone and isopropanol.^95^ Acetone and ethanol, which are both linked to diabetes, were detected via their adsorption onto the tips of nanopillars. The low limits of detection (0.0017 ng and 0.0037 ng for ethanol and acetone vapor molecules) demonstrated that the label-free, no chemical sensing approach opens the possibilities of specific and highly sensitive detection of complex VOCs in exhaled breath samples.^96^ Chen et al. developed a SERS sensor, which used reduced graphene oxide (RGO) to selectively adsorb VOC biomarkers and Au NPs that were synthesized *in situ* on the reduced graphene oxide (RGO) using hydrazine vapor.^97^ To sample, the sensor was exposed to a 500 mL breath sample in a well-sealed bag for 30 min at 37 °C, then removed and analyzed using SERS immediately to avoid biomarker desorption. Upon analysis of the SERS spectra, 14 Raman bands associated with biomarkers were selected as fingerprints to diagnose gastric cancer and distinguish between early and advanced gastric cancer patients. A SERS-based breathalyser used to distinguish VOC profiles in COVID positive individuals has been developed by Leong et al.^98^ In this approach the SERS substrate consisted of arrays of silver nanocubes functionalized with 4-mercaptobenzoate (MBA), 4-mercaptopyrdine (MPY) and 4-ATP. This is shown in Figure 4B. The multireceptor sensor interacted with VOCs via hydrogen bonding, ion-dipole interactions and π- π interactions to bring the VOCs close to the plasmonic surface. The VOCs detected included ketones, aldehydes and alcohols. The surface was analyzed using a portable Raman spectrometer, allowing for on-site analysis in 5 min. Spectral changes between positive and negative COVID breath samples were noted for each receptor, with the platform achieving a sensitivity of 96.2% and specificity of 99.9% across 501 participants. This is a crucial step in achieving noninvasive human breath diagnostics at POC.

Combining VOC detection for healthcare applications with SERS has produced a platform that can yield qualitative and quantitative information that shows promise when paired with human breath sampling to detect disease in the human body. Furthermore, when the analysis is performed using a portable spectrometer, the analysis has the capability of taking place at the POC, a positive step for healthcare applications where rapid diagnosis is vital. However, there are some challenges to overcome before it can be adopted as a routine test.^99^ This includes overcoming poor affinity of the VOCs with the SERS substrate, which is currently being investigated and achieved using various probe molecules and MOFs. Another challenge is how to incorporate substrate cooling steps into the platform, which increase sensitivity. This is attributed to the lowered desorption speed of VOCs at low temperatures. Including this step will be highly beneficial but could limit portable applications or destroy the substrate and therefore needs to be thoroughly investigated. Despite the challenges, VOC-SERS can compete with traditional methods and could be an excellent tool in breath analysis at the POC.

Most of the examples given above rely on small changes in the Raman spectra to determine if a biomarker is present. This becomes more difficult to interpret when more than one biomarker is present as the data becomes more convoluted. To deal with the complex Raman spectra obtained from these POC platforms, machine learning can be adopted to help with quantitation and discrimination.

## SERS Combined with Machine Learning for Improved Accuracy in Data Analysis

Machine learning (ML) is an area of artificial intelligence (AI) that uses data that is difficult to interpret as an input resource to yield easy-to-read results. We can see examples of its use everywhere today from innovative technology such as mobile phones and computers, to healthcare where it is used to aid disease diagnosis.^100^ Label-free SERS assays have been paired with ML to improve results, akin to that achieved by chemometrics.^101^ By applying algorithms such as PCA and partial least-squares discriminant analysis (PLS-DA) to large, complex Raman and SERS data sets, it analyses them with a higher degree of accuracy, improving biomarker recognition.^102^ This is known as unsupervised ML as it uses clustering methods and does not require labels. The label-free SERS spectra are separated based on space, where every pixel is considered a dimension. To identify what is in a cluster, some background knowledge of the sample is required. As mentioned, these techniques are very similar to chemometrics and to improve ML, more advanced algorithms have emerged that learn from data. Deep learning (DL) is a subset of ML that is based on neural networks that learn to improve accuracy. It includes techniques such as random forest (RF) and support random vector machine (SVM).^103^ DL is a supervised ML model and uses samples that contain known biomarkers to train the ML model to recognize features in the SERS spectra and assigns them labels that correspond to the biomarker. The model can then be used to detect and discriminate the biomarker in unknown samples, increasing the sensitivity and specificity of an assay. In the literature, the terms ML and DL are used interchangeably to describe how the data has been analyzed, with both being applied to SERS spectra to detect and discriminate different biomarkers and to diagnose disease.

**Figure 5:**
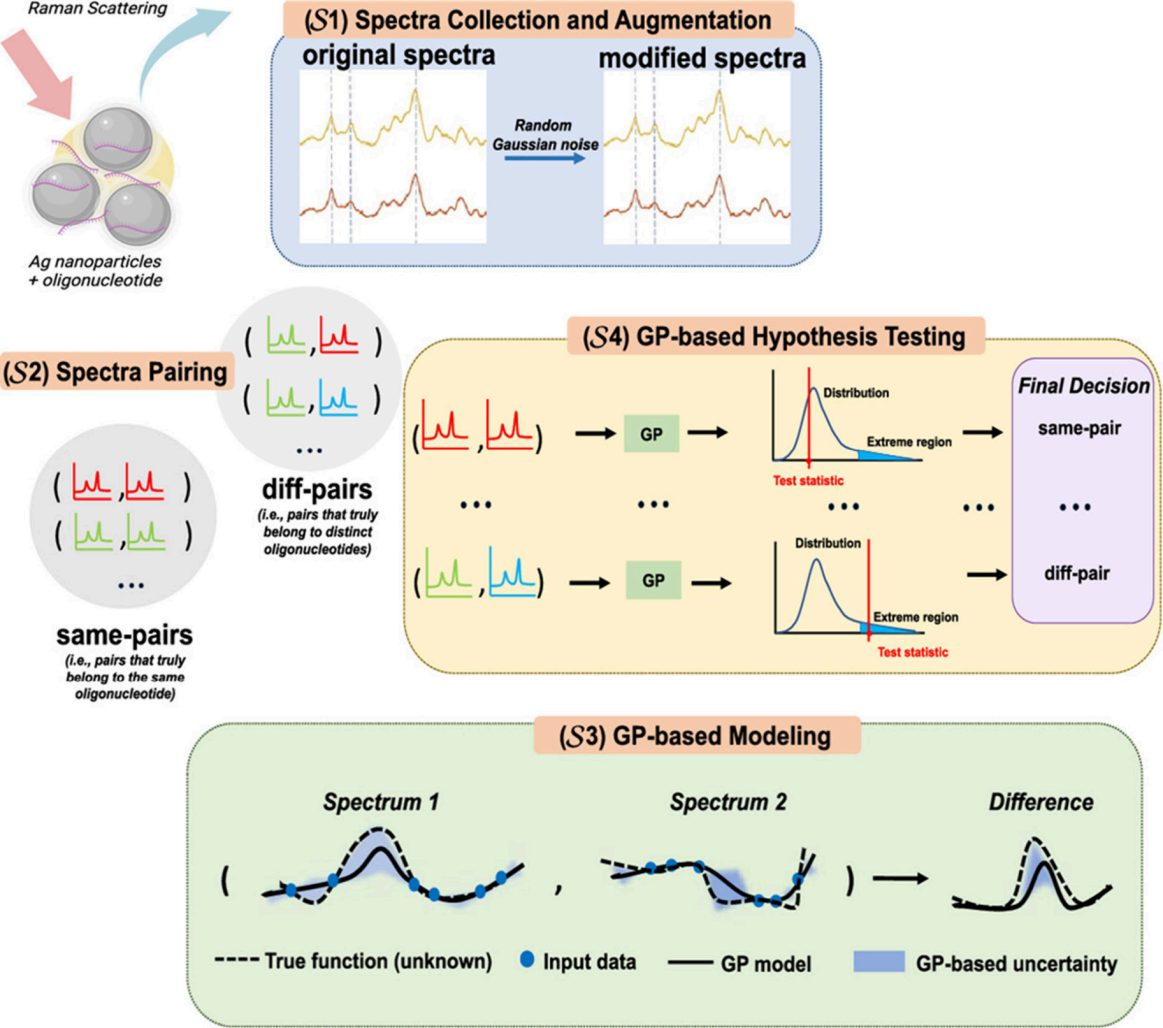
Schematic showing discrimination of DNA and RNA via SERS combined with ML. First, DNA and RNA are added to Ag NPs and the SERS signal is collected. The data are then baselined followed by a data augmentation task, where the training set is inflated by creating slightly altered versions of existing data to increase the data set. The second step forms sets of spectra pairs by matching individual spectra to form positive and negative samples for ML training and testing. In the third step the spectra data are modeled using a GP. The last step determines if a pair of spectra are different, which implies they are from different oligonucleotides.^104^ Adapted with permission from ref (104). Copyright 2024 American Chemical Society.

SERS combined with ML has been used to discriminate genetic biomarkers of disease using the label-free SERS spectra of DNA and RNA. An example of this by Chheda et al. used spermine coated Ag NPs as a positively charged SERS substrate to attract the negatively charged phosphate backbone of single stranded (ss) DNA and RNA.^104^ A series of different samples with sequence modifications, such as substitutions, additions and deletions were first analyzed followed by the analysis of the prostate cancer biomarker mir-21 and its mutated variants. To interpret the resulting SERS spectra, a functional data analysis (FDA)-based framework was developed to detect mutated DNA and RNA oligonucleotides. The framework comprised of 4 steps: 1) spectra collection and augmentation, 2) spectra pairing, 3) Gaussian process (GP)-based modeling and 4) GP-based hypothesis testing. This is shown in Figure 5. The approach accurately differentiated SERS spectra obtained from the different oligonucleotides and outperformed various data-driven methods in many metrics including accuracy, sensitivity, and specificity. The authors suggest that the combined use of SERS and ML could therefore be effective for use in disease diagnosis that could be applied for clinical applications. Nguyen et al. have also demonstrated how SERS combined with ML can be used for the compositional analysis of ssDNA. In their approach, Au and Ag nanorods were used as SERS substrates to detect differences in SERS spectra of 200-base length ssDNA molecules.^105^ A linear regression model was developed and trained along with neural network (NN) models to predict the composition of ssDNA. The results indicated that the NN model was the optimum method of analysis and mitigated effects of data dispersion that could occur due to biodegradation of samples over time or differences in SERS substrates prepared on different days. This is particularly appealing as SERS detection often struggles with reproducibility. DNA damage in spermatozoa has also been assessed using SERS and ML to validate the hypothesis that exposure to the fungicide difenoconazole reduced sperm quality.^106^ In an example by Shi et al., a SERS-based database of DNA was created which was suitable for AI based analysis and demonstrated discrimination of tumor suppressor genes.^107^ The combined technique was also able to profile DNA methylation patterns in lung cancer patients and discrimination of gene sequences.^108^ A deep learning assisted method that used SERS-based ZnO-Au direct amplification (ZADA) was developed by Kim et al. for rapid, label-free disease detection via direct nucleic acid amplification.^109^ In this example, a gold coated ZnO nanorod was used to amplify the SERS signal with nucleic acid amplification achieved via the coupling of the Au with thiol synthesized primers and the addition of recombinase polymerase amplification. Clinical validation of the test was achieved using 29 clinical samples from patients with coronavirus disease 2019 (COVID-19) and a ResNet-based learning model to predict patients with COVID-19. Overall, the platform achieved 92% sensitivity and 81% specificity with the deep learning enhancing the sensitivity and specificity, reducing false negatives and positives, and shortening the time required for SERS analysis.

SERS paired with ML has also been used to classify protein species. Barucci et al. used a silver nanowire SERS substrate onto which different protein solutions were dropped, and the SERS signal collected.^110^ The proteins investigated included human serum albumin, bovine serum albumin, lysozyme, human holo-transferrin and human apo-transferrin, which were selected due to their similar composition and/or secondary structure content. A mixed analytical approach that used PCA carried out on integrated areas of Lorentzian bands obtained by band fitting of the SERS spectra was applied and demonstrated superior classification of proteins compared to standard PCA application. Early cancer detection has been achieved via serum biomolecular fingerprinting spectroscopy and ML in an integrated method known as SERS and Artificial Intelligence for Cancer Screening (SERS-AICS).^111^ In this example, liquid biopsy samples from 382 heathy controls and 1582 patients were added to silver nanowires and the SERS measured. The SERS-AICS platform, which used a SVM model, distinguished cancer patients from healthy controls with 95.8% accuracy and 95.8% sensitivity at 95.4% specificity. The technique provides a promising comprehensive tool for real world cancer detection when used in conjunction with routine physical exams. SERS and ML has also been used for rapid diagnosis of *Mycobacterium tuberculosis* (Mtb) in sputum samples using a hand-held Raman spectrometer and deep learning algorithms, producing a platform with high potential for rapid POC detection of Mtb infection.^112^ Another example used a SERS sensing platform with controlled nanogaps and deep neural network models to discriminate the response of *Escherichia coli* and *Pseudomonas aeruginosa* to antibiotics from untreated cells in 10 min with greater than 99% accuracy.^113^ A 10-fold difference in the concentration of antibiotic dosage was also obtained when compared to conventional growth assays. The rapid discrimination of different strains of antibiotic-resistant *Klebsiella pneumoniae* has been achieved using a label-free SERS-based sensor paired with autoencoder and PCA that extracted features in a nonlinear and linear manner.^114^ The extracted features were then fed into a SVM classifier that discriminated the different strains. Another example of pairing ML with SERS has been reported by Lussier et al., who used the platform to measure gradients of metabolites *in vitro* near different cell lines.^115^ An artificial neural network (ANN) was used to extract features of the SERS spectra associated with different orientations of metabolites on the NP surface, which improved the number of metabolites detected as well as the sensitivity and selectivity. Other examples of label-free ML-SERS platforms include SERS paired with PCA-Centroid displacement nearest neighbor (CDNN) to recognize and detect precancerous lesions of gastric cancer and three-dimensional surround-enhancing SERS platforms combined with visual geometry group network for plasma exosome-based early cancer diagnosis.^116^

These examples demonstrate how ML analysis can improve the results of simple label-free SERS assays. However, they all rely on the biomarkers interacting with the SERS substrate for it to appear in the SERS signal. If a biomarker has a weak affinity for the surface, it will not be detected. Another disadvantage is that if the biomarker is in a complex matrix sample, competitive binding can occur resulting in poor detection. To increase the binding of a specific biomarker, the SERS substrate can be functionalized with a biomolecule such as an antibody, aptamer, or DNA specific to the biomarker. This brings the selected biomarker closer to the surface of the SERS substrate and it can be detected more readily via SERS. When the resulting SERS spectra are analyzed with ML, key features used for classification can be identified.

Functionalized SERS substrates have been paired with ML for the detection of Alzheimer’s disease. In one example, gold nanowires were functionalized with antibodies specific to amyloid beta or self-assembled monolayers with distinct functional groups (PMMA, methyl, carboxylic acid, or amine) to monitor different dipole interactions with blood-based metabolites.^117^ Blood plasma from Alzheimer’s patients and human controls were added to the substrates and the SERS signal collected and analyzed using a fully connected neural network classifier. Amyloid beta oligomerization was distinguished on the substrate coated in antibody demonstrating their potential in monitoring the progression of Alzheimer’s disease. The amine coated substrate had the highest accuracy for classifying human control and Alzheimer’s patients (99.5%) demonstrating that deep learning assisted SERS functionalized substrates is a promising tool at diagnosing Alzheimer’s disease. Compositional changes in culture medium arising from metabolic activity of tumor or healthy cells were detected using Au NPs grafted with various chemical moieties designed to selectively trap biomolecules of interest.^118^ This generated information-rich SERS spectra that were analyzed using convolutional neural network (CNN). The trained CNN was able to, with 100% prediction accuracy, distinguish healthy and cancer cell metabolites.

ML has also been applied to SERS assays that use the intensity of the Raman reporters bound to NPs to discriminate and/or quantify biomarkers to increase sensitivity and specificity. Banaei et al. demonstrated the rapid and purification-free detection of extracellular vesicles (EVs) from pancreatic cancer patients using a labeled SERS-based immunoassay.^119^ First, the assay captured normal and tumor derived EVs from pancreatic cancer, chronic pancreatitis, and normal control samples onto a gold substrate. The tumor derived EVs were then selectively detected using a gold SERS nanotag designed specifically to only detect the tumor EVs. The surface was analyzed using a portable Raman spectrometer and the SERS signal of the Raman reporter used to quantify biomarker expression levels. A classification tree was trained with the data set and employed to predict the condition of the patients. The sensitivity and specificity of the models were calculated as 0.95 and 0.96, respectively. SERS based LFIAs have also been paired with ML to rapidly and sensitively detect *Escherichia coli* O15:H7.^120^ The authors reported that regression models based on ML were more sensitive than traditional linear curves used for quantitative analysis. The best regression model was extreme gradient boosting regression which could solve complex prediction problems.

ML also performs well when paired with multiplexing labeled SERS assays. Li et al. synthesized seven SERS-active “nanorattles” that were loaded with different Raman reporters and applied them in a hybridization assay for the detection of multiple mRNA biomarkers for head and neck cancers.^121^ A CNN analysis was used to separate the multiplexed spectra and yielded high accuracy and fast predictions. It was then used to analyze clinical data from nonmultiplexed mRNA biomarkers using 20 patient samples and was able to identify the specific clinical biomarker with low error, demonstrating the capability of CNN-based ML for SERS-based medical diagnostics. High throughput multiplexing has been achieving using fluorescence and SERS-active nanoprobes paired with a barcode ML identification algorithm.^122^ 45 unique spectra were obtained from mixing three fluorescent and 15 Raman reporters. The spectra were transformed into a barcode using an algorithm that distinguished the spectra based on the position of all the peaks and was verified using model experiments that used the multiplexed spectra. The authors note that this barcode approach would be extremely useful for analyzing and encoding biological targets. Wang et al. have used the multiplexed SERS spectra obtained from a microdroplet-based SERS platform for the detection of EV proteins and analyzed it with ML algorithmic tools, which helped to discover the presence of different subpopulations in single-cell data sets.^123^ To understand what reporter should be selected for multiplexing, Sánchez-Purrà et al. analyzed 15 reporter molecules and used a correlation matrix to select five optimum candidates.^124^ They were used to distinguish human IgG in dipstick immunoassays with their relative contribution estimated using a non-negative least-squares (LS) algorithm. An average true positive rate (TPR) of 88% was achieved, demonstrating that the technique could be applied for the detection of nonspecific biomarkers in diverse clinical conditions.

The SERS output from VOC detection platforms have also been analyzed using ML to aid in diagnosis. For example, Li et al. was able to detect urinary volatile metabolites to diagnose phenylketonuria using a VOC sensor array with SERS measurements combined with ML analysis.^125^ The SERS-based sensor array patterned with three thiophenolic ligands, 4-ATP, 4-MBA and 4-MB, was prepared and applied for the SERS monitoring of volatile metabolites with multiplexed readouts by sampling headspace gases from the urine samples. Detection limits as low as 2 μM were achieved for phenylpyruvic acid, 4-hydroxyphenylacetic acid and phenylacetic acid, which were well below the diagnostic thresholds for phenylketonuria. The sensor was also able to perform multiplexed profiling of individual phenylketones and their mixtures at picomolar levels, and using the ML algorithms linear discriminant analysis and t-distributed stochacti neighbor embedding could discriminate those with and without phenylketonia with a diagnostic average of 97%. Another example of VOC detection using SERS and ML is by Cao et al., who applied a microfluidic silicon SERS AI chip designed for rapid preconcentration, reliable SERS detection and automatic identification of trace aldehydes at ppt levels.^126^ To discriminate SERS spectra collected from VOCs, a fully connected deep neural network containing one hidden layer with 6 neurons was used. Six distinct aldehydes were readily discriminated at low concentrations with high accuracy, laying the foundation for precise diagnosis at an early stage using VOC-SERS-ML platforms.

The accurate and sensitive discrimination of biomarkers of disease is improved by analyzing the results using ML. By applying ML models to complex SERS spectra, they can be deconvoluted and key features identified. The key features are used to identify what biomarkers are present and, in some cases, the concentration as well. The platform therefore has the potential to produce rapid and accurate results that can be used to aid healthcare professionals in decisions and treatment pathways. Of course, we should still be cautious when using ML as discussed by Masson.^127^ For example, if an improperly trained ML model is used, it will underperform, akin to using the wrong calibration curve. We should also not expect the data to be reliable or more robust just because ML has been used. Masson states that when developing a ML model, we should adhere to the 3Rs, robust, reasoning, and responsible. But most importantly, the data should always be validated.

## Conclusion and Perspective

There is potential for SERS to be applied in POC diagnostics, health and therapeutic drug monitoring and significant progress has been made. The development of portable, hand-held spectrometers has enabled SERS to be implemented at POC for rapid and sensitive detection in *in vitro* diagnostics, or in wearable sensors for real-time testing. This is a significant step in the application of SERS detection in clinical settings. In moving toward continuous monitoring and at-home testing, the capabilities of SERS-based wearable sensors have been demonstrated using various platforms and how these can be applied for the noninvasive analysis of biofluids. This is ultimately owed to the development of sophisticated nanoscale substrates with high sensitivity, stability, flexibility and biocompatibility. Wearable SERS sensors offer the sensitivity to directly detect analytes with minimal interference from biofluids, the ability to measure multiple analytes simultaneously, and the potential to monitor biofluid properties that can be associated with health conditions. Direct detection of biomolecules using wearable sensors also reduces the need for enzymes or biorecognition elements that can be unstable, costly and require additional steps. SERS is also suitable for continuous monitoring as the signal intensity varies with analyte concentration in real-time. Wearable SERS devices can be tailored to suit the specific application, and portable spectrometers can be incorporated to enable true real-time POC analysis. The development and use of SERS-based wearable sensors is an emerging area, where the greater need for personal health monitoring could potentially be met by the capabilities of SERS biosensing. However, there are still many challenges in the adoption of SERS wearables, such as costly fabrication methods, complex data analysis requirements and long-term stability of substrates or analyte signal for continuous monitoring.

SERS has also been successfully applied to VOC detection and we have highlighted the potential of the technique in this area and how it could be applied for the noninvasive analysis of breath for healthcare applications. Again, much of the success in VOC detection is owed to the development of novel substrates for analyte capture and strong SERS enhancement, which is key to the sensitivity and ability of SERS to obtain specific molecular information from low concentration samples. VOC detection using SERS has been shown to be competitive with traditional methods and could be a promising tool for POC analysis. One of the main challenges in SERS-based VOC detection is the efficient capture of the volatile molecules on SERS substrates and various methods are being explored to achieve this. Additionally, complex data interpretation is often required, which remains a challenge for VOC detection using SERS but can potentially be addressed using sophisticated data processing methods and ML. Improved accuracy in the detection of biomarkers and diagnosis of disease can be achieved when SERS is combined with ML. ML should be applied to all platforms where large data sets are generated to aid in data analysis and to increase accuracy but should be used with caution and not relied on. Sensitivity and reproducibility of SERS remains a concern; however, this can be addressed by careful substrate design, collaborative research and the use of internal standards.^128^ As discussed herein, highly scalable substrate fabrication methods have been suggested and these will help to ensure reproducibility of SERS substrates. Although the potential of SERS has been demonstrated for many POC applications, we believe that the drive toward continuous health monitoring and personalized healthcare is a key opportunity to exploit the benefits of the technique.
